# Trajectories of quality of life in pancreatic cancer survivors during the first year after surgery: a longitudinal study

**DOI:** 10.1038/s41598-026-58311-6

**Published:** 2026-06-16

**Authors:** Jinfeng Zhu, Lechun Huang, Ruixin Zheng, Zhangjie Jiang, Weishu Hu, Jing Cao, Junfeng Zhang, Li Tang, Rong Huang

**Affiliations:** Chongqing Academy of Medical Sciences, Chongqing General Hospital, Chongqing University, Chongqing, 401147 China

**Keywords:** Pancreatic cancer, Cancer survivors, Longitudinal study, Quality of life, Cancer, Gastroenterology, Oncology

## Abstract

Although quality of life (QOL) improves over time for most pancreatic cancer patients after surgery, some patients may show different patterns of QOL. Beyond determining distinct QOL trajectories, identifying characteristics of patients who have different trajectories can help identify pancreatic cancer patients who may benefit from intervention. We aimed to identify trajectories of QOL in pancreatic cancer patients for one year after the surgery, to determine the factors influencing these changes. This longitudinal study recruited 120 pancreatic cancer patients, and their QOL, anxiety, depression, social support, self‑efficacy, and sleep quality were assessed. Data were collected at 1 month (T1), 3month (T2), 6month (T3), and 12month (T4) after surgery. Group-Based Multivariate Trajectory Modeling (GBMTM) was used to identify distinct subgroups of patients with similar patterns of QOL change after surgery. A univariate analysis was used to determine which variables were associated with trajectory membership. A multinomial logistic regression was performed to identify factors associated with trajectory group membership. 104 patients were analyzed and identified with three trajectory groups, the low, moderate and high QOL group. All of three groups exhibited a sustained upward trend in functional domain scores, followed by a slight decline at 12 months. Correspondingly, symptom domain scores showed a consistent downward trajectory across all groups, with a slight increase observed at 12 months. Compared with the high QOL group, the odds of the low QOL group were 1.139 times higher in the SDS (OR = 1.139, 95% CI: 1.037–1.252, *P* = 0.007), and the odds of the low QOL group were 1.762 times higher than the high QOL group in the PSQI (OR = 1.762, 95% CI: 1.161–2.674, *P* = 0.008). Compared to the high QOL group, the odds of the moderate QOL group were 1.127 times higher in the SDS (OR = 1.127, 95% CI: 1.037–1.226, *P* = 0.005), and the odds of the moderate QOL group were 1.535 times higher in the PSQI (OR = 1.535, 95% CI: 1.055–2.235, *P* = 0.025). Conversely, occupation, BMI, SAS scores, and GSES scores showed no statistically significant effect on trajectory group (*P* > 0.05). Identifying high‑risk groups for reduced QOL after surgery is necessary. Moreover, psychosocial interventions and sleep methodologies should be provided to alleviate psychological symptoms and improve sleep quality to enhance patients’ QOL.

**Supplementary Information:**

The online version contains supplementary material available at https://doi.org/10.1038/s41598-026-58311-6.

## Background

 Pancreatic cancer is one of the most lethal cancers and increasing worldwide, ranking tenth among cancers occurring in China^1,2^. Despite the survival rate and survival time of pancreatic cancer patients have been increasing with the development of screening, neoadjuvant therapies and surgery in the past few decades^3^. However, it was reported that the 5-year survival rate worldwide has had minimal increase over the last 30 years. Moreover, with the rapidly development of national economic, patients live for a longer time as pancreatic cancer survivors after the surgery^4^. The survival time is no longer the only endpoint evaluation indicators for the effective treatment, and the improvement of quality of life (QOL) after surgery is now considered equally important to survival time^5^.

The first year after surgery represents a critical period for physical recovery, complication management, adjuvant therapy, and psychological adjustment, during which quality of life may exhibit complex, non-linear trajectories^6,7^. The successful completion of surgery after neoadjuvant treatment is the beginning of the transition from cancer patient to cancer survivor^8^. After surgery, patients with pancreatic cancer can gradually return to their former live^9^. However, during this postoperative period, patients with pancreatic cancer may experience varying degrees of long-term physical, psychological, and social distress, which complicates survivorship and presents substantial challenges^10^. Although some cancer-related concerns tend to decrease with time, many pancreatic cancer patients face physical (physical activity, fatigue, pain, sleep disturbance), psychological (anxiety, depression, fear, low self-confidence), and social (avoidance, re-employment) problems related to the sequelae of treatment^11^. These problems reduce adaptability and QOL in cancer survivors who have to learn and adapt to “Living with Cancer,” and pose a significant challenge in the recovery process^12^.

Besides, owing to the organ’s essential role in digestion, absorption, and blood glucose regulation, changes in the QOL of patients after pancreatic surgery have attracted the attention of surgeons^13^. However, the QOL after surgery for pancreatic cancer patients is influenced by multiple dimensions: physiologically, they often face long-term challenges such as pancreatic endocrine and exocrine insufficiency, chronic pain, and severe fatigue; psychologically, they commonly experience high levels of anxiety, depression, and fear of recurrence; socially, they frequently encounter role dysfunction and difficulties in reintegrating into society^14,15^. What’s worse, it is challenging to follow up the QOL of patients once they are discharged from the hospital^16^.

To identify the duration and components of an intervention program that supports the transition from cancer patients to survivors, empirical evidence needs to be accumulated through an integrated investigation of changes in the QOL of pancreatic cancer patients and the factors affecting these changes. In previous studies, factors affecting the QOL of pancreatic cancer survivors included physical and psychological symptoms, self-efficacy, and social support^7,15,17,18^. The greater the symptoms experienced by pancreatic cancer patients, the higher the psychological distress level, the lower their physical and social functioning, and the worse the over-all QOL^19,20^. Self-efficacy positively affects the QOL of pancreatic cancer survivors by helping them actively cope with cancer-related health problems and maintaining self-management and health behavior for various symptoms^21^. Social support is also a representative environmental factor affecting the QOL of pancreatic cancer patients; the provision of sufficient social support, such as feeling protected or receiving help from others, assists survivors in actively coping with health problems and finding positive meaning in life, which ultimately improves their QOL^7^.

Although a number of studies have examined QOL outcomes in patients with pancreatic cancer, few have investigated postoperative QOL trajectories, and most existing studies have been limited by small sample sizes or insufficient long-term follow-up results^22–24^. Moreover, existing studies predominantly employ cross-sectional designs, assessing quality of life at a single time point, which fails to capture its dynamic trajectory^24^. Furthermore, studies on influencing factors are often confined to single dimensions, lacking systematic analysis of multiple factors including demographics, clinical treatment, and psychosocial aspects^23^.

Besides, longitudinal studies and trajectory analyses facilitate more individualized patient care. Management strategies informed by these approaches can guide the development of targeted and realistic interventions. In addition, identifying baseline determinants of QOL trajectories, referred to as baseline trajectory models, enables the design of timely interventions to improve patients’ subsequent QOL^25^. Therefore, there is an urgent need for longitudinal research to systematically characterize early postoperative QOL trajectories among patients with pancreatic cancer and to elucidate their multidimensional determinants. The aim of this study was to explore the trajectory of QOL in pancreatic cancer patients and which factors predict the different trajectories.

## Methods

### Study design and participants

This study employed a longitudinal design to examine trajectories of quality of life (QOL) in patients with pancreatic cancer over the first postoperative year. Participants were 120 patients recruited via purposive sampling following pancreatectomy at Chongqing General Hospital, Chongqing University, China. Eligible participants met the following criteria: (1) age between 19 and 79 years; (2) histologically confirmed stage I or II pancreatic cancer, status post-pancreatectomy and hospital discharge; (3) no evidence of cancer recurrence or metastasis; (4) proficiency in Chinese with the ability to complete self-report questionnaires; and (5) current receipt of adjuvant chemotherapy or targeted therapy for recurrence prevention. Participants were excluded if they met any of the following criteria: (1) a diagnosed psychiatric disorder, including but not limited to adjustment disorder, obsessive-compulsive disorder, or anxiety disorder, or current use of associated pharmacological agents; or (2) preexisting medical conditions that could contribute to cognitive impairment, such as stroke or dementia.

Participants were recruited from the hospital’s patient registry between January 2024 and August 2025. Pancreatic cancer patients who satisfied the eligibility criteria and provided written informed consent were included in the study. Assessments were conducted at four time points post-surgery: one month (T1), three months (T2), six months (T3), and twelve months (T4).

With a significance level of 0.05, a minimum important difference (MID) of 6 points on the PAN-26 functional and symptom domains, and a power of 0.80, G*Power analysis indicated that 89 participants were required^26^. Accounting for a 30% dropout rate, 120 patients were enrolled.

### Study instrument

#### Quality of life

Cancer-specific health-related quality of life (HRQL) was evaluated using the European Organization for Research and Treatment of Cancer (EORTC) Core Quality of Life Questionnaire (QLQ-C30)^24^. The questionnaire consisted of 30 items, which yielded the following subscales: five multi-item functioning scales (physical, role, social, emotional, and cognitive); three multi-item symptom scales (fatigue; nausea and vomiting; and pain); six single-item symptom scores (dyspnea, insomnia, appetite loss, constipation, diarrhea, and financial difficulties); and a two-item global quality of life (QOL) scale. Responses were recorded on a four-point Likert scale (ranging from “not at all” to “very much”), except for the two global QOL items, which used a seven-point Likert scale. Raw scores were linearly transformed to a 0–100 scale. Overall QOL was assessed using the summary score, defined as the mean of all QLQ-C30 subscales except for the global QOL and financial difficulties items. A higher summary score corresponded to a better overall QOL.

The QLQ-PAN26 was developed as a supplementary module to the QLQ-C30, specifically designed for use in patients with pancreatic disease. Validation studies had confirmed its reliability in patients who had undergone pancreatic surgery, demonstrating its ability to detect clinically important differences after pancreatectomy^27^. The instrument consisted of 26 items, which were grouped into two domains: nine scales measuring disease-related symptoms and treatment side effects (pancreatic pain, eating-related problems, altered bowel habit, hepatic symptoms, side effects, cachexia, indigestion, ascites, and flatulence), and five emotional scales or items specific to pancreatic cancer (body image, healthcare satisfaction, sexuality, fear of future health, and ability to plan for the future). Scale scores were linearly transformed to a range of 0 to 100. For both instruments, higher scores on global health status and functioning or emotional scales denoted better HRQL and higher levels of functioning, whereas higher scores on symptom scales denoted greater symptom severity and consequently poorer HRQL.

### Symptom experience

#### The Pittsburgh sleep quality index (PSQI)

Subjective sleep quality was assessed using the Pittsburgh Sleep Quality Index (PSQI), developed by Buysse et al.^28^. Each component was rated on a 0–3 scale, with higher scores indicating greater severity (0 = no problem; 3 = very severe problem). The total score, which ranged from 0 to 21, was derived by summing the component scores. A total score of ≤ 7 was conventionally interpreted as indicative of good sleep quality, whereas scores above this threshold denoted progressively poorer sleep quality.

#### The self-rating anxiety scale (SAS)

Anxiety symptoms were evaluated using the Self-Rating Anxiety Scale (SAS), developed by Zung et al.^29^. The instrument consisted of 20 items that assessed the frequency of anxiety-related symptoms, each rated on a 4-point Likert scale: 1 (none or very little of the time), 2 (some of the time), 3 (a considerable amount of the time), and 4 (most or all of the time). Raw scores were transformed according to standardized procedures to obtain a total score, with higher scores reflecting more severe anxiety. Clinical interpretation was based on the following cutoffs: <50, no anxiety; 50–59, mild anxiety; 60–69, moderate anxiety; and ≥ 70, severe anxiety.

#### The self-rating depression scale (SDS)

Depressive symptoms were evaluated using the Self-Rating Depression Scale (SDS), developed by Zung et al.^30^. The instrument comprised 20 items, each scored on a 4-point scale. Total raw scores were converted to a standardized index score, with higher scores denoting more severe depression. Clinical interpretation was based on the following cutoffs, < 53, no depression; 53–62, mild depression; 63–72, moderate depression; and ≥ 73, severe depression. The SDS was notable for its ease of administration and its capacity to directly capture patients’ subjective depressive experiences, with established validity across age, sex, and socioeconomic strata.

### Social support

#### The social support rating scale (SSRS)

Social support was assessed using the Social Support Rating Scale (SSRS), developed by Xiao^31^. The instrument consisted of 10 items measuring three dimensions: subjective support, objective support, and utilization of support. Each item was scored on a 4-point scale, with higher scores reflecting greater social support. The scale had demonstrated sound psychometric properties, with Cronbach’s alpha coefficients between 0.825 and 0.896, inter-subscale correlations ranging from 0.462 to 0.664, and subscale-total correlations between 0.724 and 0.835^31^.

#### The general self-efficacy scale (GSES)

The General Self-Efficacy Scale (GSES), developed by Schwarzer and Jerusalem in 1981, assessed an individual’s self-perceived ability to cope with difficulties and had been established as a predictor of behavioral choices^32^. In this study, the GSES was used to evaluate participants’ overall self-efficacy when encountering frustration or adversity. The scale comprised 10 items, each rated on a 4-point Likert scale ranging from 1 (“completely incorrect”) to 4 (“completely correct”), yielding total scores between 10 and 40. Higher scores reflected greater self-efficacy. The Chinese version of the scale used in this study demonstrated satisfactory reliability, with a Cronbach’s alpha coefficient of 0.870.

### Data collection

Data collection occurred from January 2024 to August 2025. Postoperative assessments and follow-up were conducted in person at the outpatient clinic during routine follow-up visits or during inpatient hospitalizations for chemotherapy. When in-person follow-up was not possible, data were collected via telephone or online platforms. Clinical data were abstracted from electronic medical records using a standardized case report form. Each participant was assigned a unique study identifier to ensure data confidentiality and compliance with the Privacy Policy. A nominal incentive was provided to participants at each data collection time point.

All outcomes related to the scales used in this study were based entirely on questionnaire-based patient-reported outcome (PRO) assessments. No interviews were conducted. All participants independently completed the self-administered questionnaires based on their own subjective perceptions and experiences.

### Data analysis

Baseline characteristics were described based on the cleaned, complete dataset, with summary statistics presented in baseline tables. Categorical variables were reported as frequencies and percentages, whereas continuous variables were expressed as mean ± standard deviation ($\:\stackrel{\mathrm{̄}}{\mathrm{x}}\:$± s) or median with interquartile range, depending on the distribution. Intergroup comparisons were conducted using independent samples t-tests or Wilcoxon signed-rank tests for continuous variables, and Pearson’s chi-square tests or Fisher’s exact tests for categorical variables.

#### Trajectory model analysis

Scores for quality of life across functional and symptomatic domains were converted to Z-scores for standardization. Multivariate trajectory models were fitted to identify the optimal number and shape of trajectory groups. Model selection was guided by evaluation metrics including the Akaike Information Criterion (AIC), Bayesian Information Criterion (BIC), average posterior probability, entropy, and category probabilities, with the model exhibiting optimal fit and classification performance being selected.

#### Univariate analysis

Between-group differences in demographic characteristics, clinical variables, and scale scores were compared across trajectory groups identified by the multivariate trajectory model (GBMTM). For continuous variables, one-way analysis of variance (ANOVA) was used when normality and homogeneity of variance assumptions were satisfied; the Kruskal–Wallis test was applied when these assumptions were violated. For categorical variables, comparisons were conducted using the chi-square test for two-group analyses and Fisher’s exact test for analyses involving more than two groups.

#### Multivariate analysis

Multinomial logistic regression was used to identify factors associated with trajectory group membership. The dependent variable was trajectory group classification, and independent variables included all factors with a univariate P-value < 0.1. One trajectory group served as the reference category against which the other groups were compared. The influence of each independent variable on trajectory group membership was assessed using odds ratios (ORs) with corresponding 95% confidence intervals (CIs).Subgroup analyses were pre-specified as exploratory. Due to limited sample sizes in certain subgroups (e.g., occupational categories), these analyses lacked sufficient power for definitive hypothesis testing. Results are presented as hypothesis-generating and should be interpreted with appropriate caution.

Statistical analyses were performed using R Studio (version 4.5.1). All tests were two-tailed, and a P-value threshold of less than 0.05 was considered statistically significant.

### Ethical considerations

Ethical approval for this study was obtained from the Institutional Review Board of Chongqing General Hospital, Chongqing University (approval No. KYS2024-064-01). The study was conducted in compliance with the principles outlined in the Declaration of Helsinki. Before data collection, a research assistant explained the study’s purpose, content, ethical safeguards, and data collection procedures to patients during outpatient visits. Written informed consent was obtained from all participants prior to enrollment.

## Results

### General demographic characteristics

The sociodemographic characteristics of pancreatic cancer patients in this study are presented in Table 1. A total of 120 pancreatic cancer patients were recruited in the study, but 16 patients were lost to follow up due to death, withdrawal and out of contact. Eventually,104 patients completed all follow-up assessments, comprising 64 male patients (61.54%) and 40 female patients (38.46%). Regarding age distribution, the majority were aged 59 years or younger (56 cases, 53.85%); 24 patients (23.08%) were aged 60–70 years, while 24 patients (23.08%) were aged 71 years or older, with the latter two age groups accounting for an identical proportion. In terms of Body Mass Index (BMI), the highest proportion comprised patients of normal weight (67 cases,64.42%), underweight patients numbered 27 (25.96%), overweight patients were the least numerous, at 10 cases (9.62%). Occupationally, retired patients formed the primary group, comprising 66 cases (63.46%); office workers accounted for 19 cases (18.27%), and freelancers for 9 cases (8.65%), civil servants and other occupations each comprised 5 cases (4.81%).When categorizing surgical procedures into four types, Pancreaticoduodenectomy(PD) was most common with 41 cases (39.42%),Laparoscopic Pancreaticoduodenectomy (LPD) had 27 cases (25.96%), Laparoscopic Distal Pancreatectomy (LDP) had 20 cases (19.23%), Distal Pancreatectomy (DP) was the least common with 16 cases (15.38%). Regarding educational attainment, patients with junior secondary education or below constituted the largest proportion at 44 cases (42.31%), senior secondary education comprised 30 cases (28.85%), postgraduate education or above accounted for 17 cases (16.35%), university education was the least common at 13 cases (12.5%). Regarding medical insurance coverage, employee medical insurance provided the widest coverage, encompassing 79 cases (75.96%), non-local medical insurance covered 25 cases (24.04%). When categorizing surgical approaches into two types, PD (LPD) comprised 68 cases (65.38%), DP (LDP) comprised 36 cases (34.62%).

**Table 1:** Demographic data description (*N* = 104).

Variable	Description (*n* = 104)
Sex, n (%)	
Male	64(61.54)
Female	40 (38.46)
Age Group, year, n (%)	
~ 59	56 (53.85)
60 ~ 70	24 (23.08)
71~	24 (23.08)
BMI, n (%)	
Underweight	27 (25.96)
Normal weight	67 (64.42)
Overweight	10 (9.62)
Occupation, n (%)	
Civil servant	5 (4.81)
Retired	66 (63.46)
Staff	19 (18.27)
Freelancer	9 (8.65)
Others	5 (4.81)
Surgical Method, n (%)	
PD	41 (39.42)
LDP	20 (19.23)
LPD	27 (25.96)
DP	16 (15.38)
Educational attainment, n (%)	
Junior High School and Below	44 (42.31)
Senior High School	30 (28.85)
University	13 (12.5)
Postgraduate and above	17 (16.35)
Type of medical insurance, n (%)	
Employee medical insurance	79 (75.96)
Non-local medical insurance	25 (24.04)
Surgical Method2, n (%)	
PD&LPD	68 (65.38)
DP&LDP	36 (34.62)

Frequency (percentage) is used to describe the grade data.

### The scores for each scale at different follow-up time points

The scores for each scale at the four postoperative follow-up time points (1, 3, 6, and 12 month) in this study were presented in Table 2. Results indicated that for the functional and symptom domains of the pancreatic cancer QOL scale. The functional domain scores increased from 61.48 ± 17.57 at 1 month after surgery to 81.93 ± 14.09 at 6 month, before declining to 78.65 ± 19.26 at 12 month, demonstrating statistically significant differences (*P* < 0.001). The symptom domain scores decreased from 32.91 ± 14.67 at 1 month to 15.06 ± 12.71 at 6 month, before rising to 18.37 ± 16.20 at 12 month, with statistically significant differences (*P* < 0.001). The SAS scores remained within the anxiety-free range (< 50 points) throughout the study period, decreasing from 44.10 ± 8.37 at 1 month to 36.53 ± 6.88 at 6 month (the lowest point), before slightly increasing to 37.94 ± 10.47 at 12 month. These differences were statistically significant (*P* < 0.001). The SDS scores remained within the non-depressed range (< 53 standardized points) throughout the study period, with scores of 44.81 ± 10.36, 46.67 ± 11.63, 45.14 ± 11.74, and 46.25 ± 12.15 from at 1 month to 12 month respectively, showing no statistically significant difference (*P* = 0.604). The SSRS score remained stable throughout the study period with no significant fluctuations, which from 1 month to 12 month were 39.21 ± 6.87, 39.45 ± 6.79, 39.34 ± 6.66, and 39.44 ± 6.67 respectively, showing no statistically significant differences (*P* = 0.993); The GSES scores (range 10–40 points) increased from 23.27 ± 5.50 at 1 month to 27.11 ± 5.40 at 6 month, before declining to 25.60 ± 6.41 at 12 month, representing a statistically significant difference (*P* < 0.001). The PSQI scores remained above 7 points (indicating poor sleep quality) throughout the study period, decreasing from 12.06 ± 2.60 1 month to 10.42 ± 2.40 at 6 month, before rising again to 10.81 ± 2.45 at 12 month. These differences were statistically significant (*P* < 0.001).

**Table 2:** Scores for each dimension of the scale (*n* = 104).

Score	Postoperative follow-up time				Statistic	*P*
	1 month (*n* = 104)	3 month (*n* = 104)	6 month (*n* = 104)	12 month (*n* = 104)		
Functional domain, Mean ± SD	61.48 ± 17.57	74.24 ± 15.30	81.93 ± 14.09	78.65 ± 19.26	F = 30.080	**< 0.001**
Symptom domain, Mean ± SD	32.91 ± 14.67	22.76 ± 14.67	15.06 ± 12.71	18.37 ± 16.20	F = 29.306	**< 0.001**
Anxiety, Mean ± SD	44.10 ± 8.37	39.86 ± 7.51	36.53 ± 6.88	37.94 ± 10.47	F = 15.899	**< 0.001**
Depression, Mean ± SD	44.81 ± 10.36	46.67 ± 11.63	45.14 ± 11.74	46.25 ± 12.15	F = 0.617	0.604
Social support All, Mean ± SD	39.21 ± 6.87	39.45 ± 6.79	39.34 ± 6.66	39.44 ± 6.67	F = 0.029	0.993
Self-efficacy, Mean ± SD	23.27 ± 5.50	25.75 ± 5.16	27.11 ± 5.40	25.60 ± 6.41	F = 8.298	**< 0.001**
PSQI, Mean ± SD	12.06 ± 2.60	10.93 ± 2.53	10.42 ± 2.40	10.81 ± 2.45	F = 8.284	**< 0.001**

Mean ± SD; F: ANOVA.

### The QOL trajectory types and characteristics after pancreatic cancer surgery

**Fig. 1:**
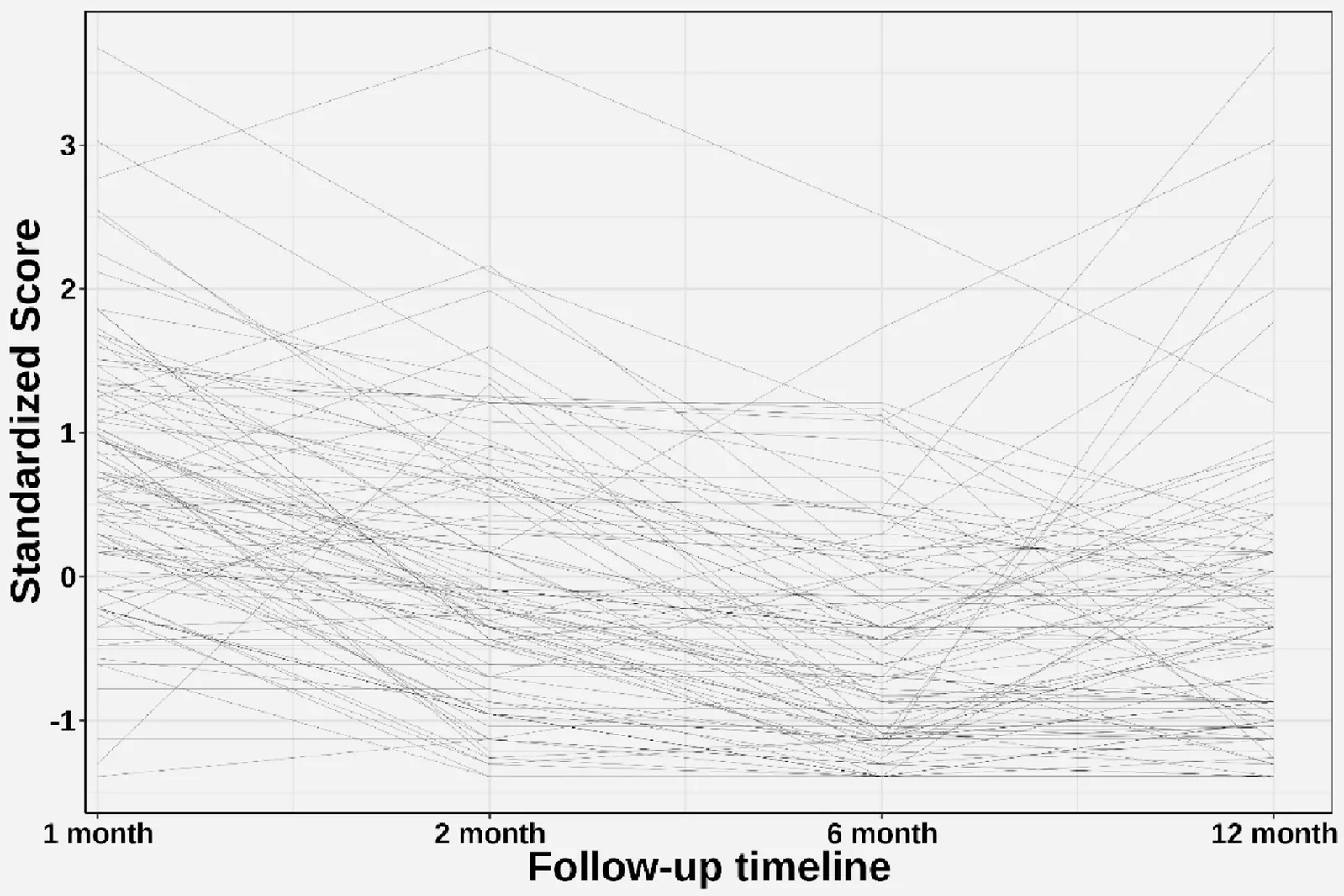
Overall change curve in patient functional domain scores(*n* = 104).

**Fig. 2:**
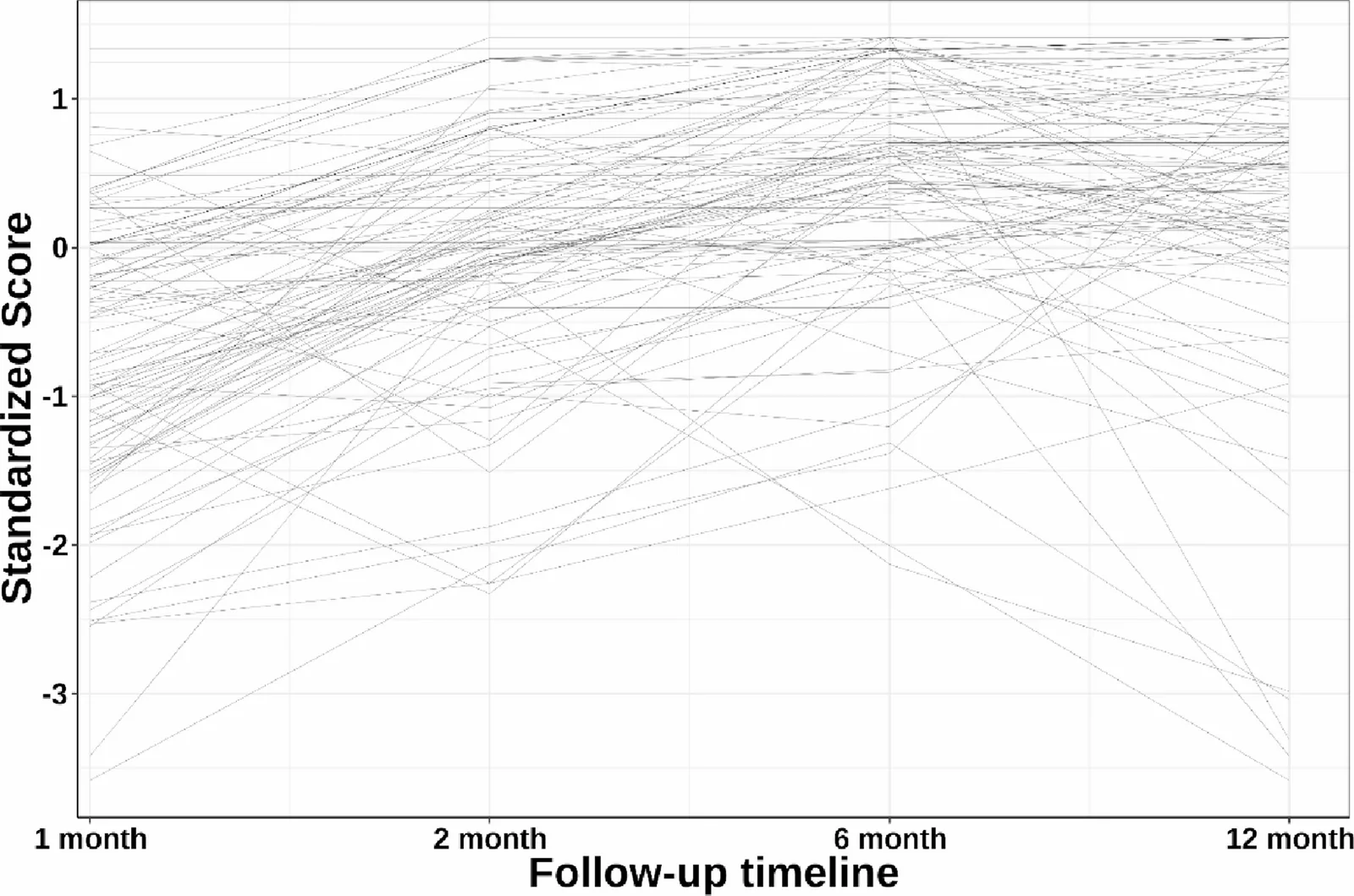
Overall change curve in patient symptom domain scores (*n* = 104). The scores of functional domain and symptom domain were standardized by Z-scores. Higher standardized scores in the functional domain indicate better quality of life, while lower standardized scores in the symptom domain indicate better quality of life.

The trajectory curves of standardized scores in functional and symptom domains for each individual were respectively illustrated (Figs. 1 and 2). It was readily apparent that scores within both functional and symptom domains exhibit significant heterogeneity among pancreatic cancer patients.

Considering both functional domain scores and symptom domain scores as variables, a group-based multivariate trajectory model (GBMTM) was employed to explore the QOL trajectory models in the participants. Eventually, our study adopted a three-category, bivariate trajectory model fitted with a non-linear curve containing quadratic terms as the optimal trajectory model, as detailed in Table 3.

**Table 3:** Effectiveness of multivariate trajectory model fitting for standardized function, symptom scores.

ng	AIC	BIC	Ek	Avepp					Class Proportion(%)				
				1	2	3	4	5	1	2	3	4	5
Linear													
2	1629.16	1689.62	0.82	0.96	0.92				75.96	24.04			
3	1565.36	1658.07	0.81	0.91	0.93	0.94			49.04	32.69	18.27		
4	1535.62	1656.54	0.78	0.94	0.93	0.88	0.93		59.62	19.23	12.5	8.65	
5	1533.6	1686.76	0.82	0.94	0.91	0.9	0.94	0.99	56.73	20.19	12.5	4.81	5.77
Quadretic													
2	1597.86	1674.44	0.84	0.96	0.93				75.96	24.04			
3	1496.8	1613.69	0.81	0.92	0.95	0.92			23.08	49.04	27.88		
4	1483.85	1632.98	0.77	0.96	0.9	0.92	0.89		54.81	21.15	14.42	9.62	
5	1484.8	1678.27	0.86	0.94	0.91	0.94	0.97	1	55.77	20.19	14.42	5.77	3.85

ng: number of trajectory group sets; d:1 means linear function, 2 means nonlinear function with square term; AIC, BIC: Akaike Information Criterion, Bayesian Information Criterion, the smaller means the better the model fitting; Ek: entropy value, the larger means the better the quality of trajectory classification; Class Proportion.

**Fig. 3:**
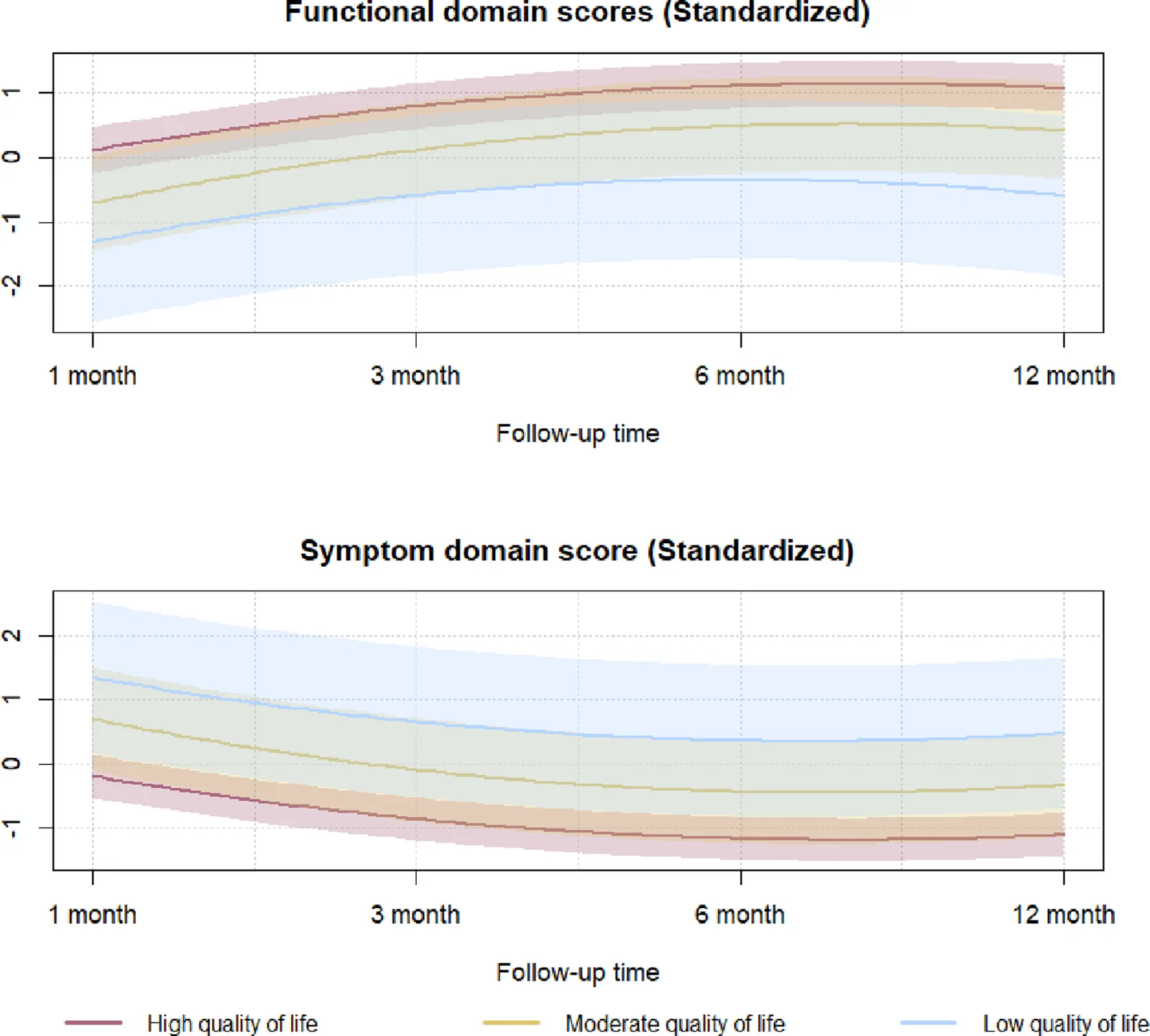
Trends in function, symptom scores (standardized) for the three different trajectories(*n* = 104). Group 1: High quality of life group; Group 2: Moderate quality of life group; Group 3: Low quality of life group; The shaded area represents the 95% confidence interval for each trajectory group score; The scores of functional domain and symptom domain were standardized by Z-scores.Higher standardized scores in the functional domain indicate better quality of life, while lower standardized scores in the symptom domain indicate better quality of life.

We modelled the QOL trajectory patterns for standardized scores in functional and symptom domains among the participants. Based on the trajectory patterns of the two domains, there were three groups named as follows, Group 1 exhibited high functional domain scores and low symptom domain scores, termed the High QOL Group, Group 2 exhibited slightly lower functional domain scores and slightly higher symptom domain scores than the High QOL Group, termed the Moderate QOL Group, Group 3 demonstrated the lowest functional domain scores and highest symptom domain scores, termed the Low QOL Group. As a result, the QOL of the participants showed a trend of continuous improvement, with a slight decline at 12month (Fig. 3).

### Identifying important determinants of QOL trajectory patterns

The univariate analysis revealed that there were statistically significant differences among the three trajectory groups in terms of BMI distribution, occupational type, anxiety scores, depression scores, self-efficacy scores, and PSQI scores (all *P* < 0.05). The low QOL group consistently performed the worst in terms of psychological and sleep status, while differences in the remaining indicators among the different trajectory groups were not statistically significant (*P* > 0.05), as detailed in Table 4.

**Table 4:** Comparison of demographic characteristics, scores on each scale in the QOL trajectory model.

Variable	Total(*n* = 104)	Traj Group			Statistic	*P*
		High QOL group(*n* = 24)	Moderate QOL group(*n* = 51)	Low QOL group(*n* = 29)		
Sex, n (%)						
Male	64(61.54)	14 (58.33)	31 (60.78)	19 (65.52)	χ²=0.310	0.856
Female	40 (38.46)	10 (41.67)	20 (39.22)	10 (34.48)		
Age Group, year, n (%)						
~ 59	56 (53.84)	12 (50.00)	29 (56.86)	15 (51.72)	χ²=4.997	0.288
60 ~ 70	24 (23.08)	9 (37.50)	10 (19.61)	5 (17.24)		
71~	24 (23.08)	3 (12.50)	12 (23.53)	9 (31.04)		
BMI, n (%)						
Underweight	27 (25.96)	5 (20.83)	9 (17.65)	13 (44.83)	–	**0.004**
Normal weight	67 (64.42)	13 (54.17)	40 (78.43)	14 (48.28)		
Overweight	10 (9.62)	6 (25.00)	2 (3.92)	2 (6.90)		
Occupation, n (%)						
Civil servant	5 (4.81)	2 (8.33)	1 (1.96)	2 (6.90)	–	**0.041**
Retired	66 (63.46)	13 (54.17)	33 (64.71)	20 (68.97)		
Staff	19 (18.27)	6 (25.00)	9 (17.65)	4 (13.79)		
Freelancer	9 (8.65)	1 (4.17)	8 (15.69)	0 (0.00)		
Others	5 (4.81)	2 (8.33)	0 (0.00)	3 (10.34)		
Surgical method, n (%)						
PD	41 (39.42)	10 (41.67)	17 (33.33)	14 (48.28)	–	0.361
LDP	20 (19.23)	6 (25.00)	11 (21.57)	3 (10.34)		
LPD	27 (25.96)	4 (16.67)	13 (25.49)	10 (34.48)		
DP	16 (15.38)	4 (16.67)	10 (19.61)	2 (6.90)		
Educational attainment, n (%)						
Junior High School and below	44 (42.31)	8 (33.33)	25 (49.02)	11 (37.93)	–	0.738
Senior High School	30 (28.85)	9 (37.50)	13 (25.49)	8 (27.59)		
University	13 (12.5)	2 (8.33)	6 (11.76)	5 (17.24)		
Postgraduate and above	17 (16.35)	5 (20.83)	7 (13.73)	5 (17.24)		
Type of medical insurance, n (%)						
Employee medical insurance	79 (75.96)	17 (70.83)	40 (78.43)	22 (75.86)	χ²=0.516	0.773
Non-local medical insurance	25 (24.04)	7 (29.17)	11 (21.57)	7 (24.14)		
Recurrence status						
No	75(72.12%)	18(75.00%)	39(76.47%)	18 (62.07%)	χ²=2.036	0.361
Yes	29(27.88%)	6(25.00%)	12(23.53%)	11 (37.93)		
Surgical Method2, n (%)						
PD&LPD	68 (65.38)	14 (58.33)	30 (58.82)	24 (82.76)	χ²=5.365	0.068
DP&LDP	36 (34.62)	10 (41.67)	21 (41.18)	5 (17.24)		
Anxiety, Mean ± SD	44.10 ± 8.37	38.91 ± 5.88	44.14 ± 7.32	48.32 ± 9.58	H = 14.748	**< 0.001**
Depression, Mean ± SD	44.81 ± 10.36	37.50 ± 7.59	46.72 ± 9.92	47.50 ± 10.59	F = 9.030	**< 0.001**
Social support, Mean ± SD	39.21 ± 6.87	39.96 ± 7.13	38.82 ± 7.31	39.28 ± 5.99	F = 0.221	0.802
Self-efficacy, Mean ± SD	23.27 ± 5.50	26.92 ± 4.83	22.37 ± 5.24	21.83 ± 5.31	F = 7.878	**< 0.001**
PSQI, Mean ± SD	12.06 ± 2.60	10.29 ± 1.47	12.11 ± 2.51	13.43 ± 2.67	H = 20.855	**< 0.001**

Mean ± SD; F: ANOVA, $\:\chi\:$²: Chi-square test, -: Fisher exact, H: Kruskal-Wallis H test; including baseline demographic characteristics, surgery-related information, and scores on each scale in the first postoperative month.

The multinomial logistic regression analysis showed that, compared with the high QOL group, a 1-point increase in the depression scale score was associated with a significantly higher probability of being classified into the moderate QOL group (OR = 1.127, 95% CI: 1.037–1.226, *P* = 0.005) and the low QOL (OR = 1.139, 95% CI: 1.037–1.252, *P* = 0.007). Similarly, for every 1-point increase in the PSQI score, the odds of being classified into the moderate QOL group increased significantly (OR = 1.535, 95% CI: 1.055–2.235, *P* = 0.025) as well as into the low QOL group (OR = 1.762, 95% CI: 1.161–2.674, *P* = 0.008). No significant effects were observed for occupation, BMI, anxiety scores, or self-efficacy scores. Notably, complete data separation occurred in two occupational categories, there is no self-employed individuals were classified into the low QOL group, and no individuals in the Others occupational category were classified into the moderate QOL, which resulting in outlier OR and P values (Table 5).

**Table 5:** Determinants of different quality-of-life trajectory patterns.

Variable	low QOL group(*n* = 29)					moderate QOL group(*n* = 51)				
	β	*P*	OR	95%CI		β	*P*	OR	95%CI	
BMI(Reference: Normal weight)										
Underweight	1.006	0.282	2.735	0.437	17.116	-0.622	0.473	0.537	0.098	2.940
Overweight	-0.283	0.824	0.754	0.062	9.088	-1.179	0.340	0.307	0.027	3.462
Occupation, (Reference: Civil Servant)										
Retired	-0.345	0.845	0.708	0.022	22.733	2.953	0.086	19.170	0.659	557.480
Staff	-2.972	0.141	0.051	0.001	2.668	1.132	0.539	3.102	0.084	115.161
Freelancer	-16.492	**< 0.001**	< 0.001	< 0.001	< 0.001	2.689	0.198	14.710	0.246	879.573
Others	-1.353	0.537	0.258	0.004	18.965	-14.623	**< 0.001**	< 0.001	< 0.001	< 0.001
Surgical method, (Reference: PD)										
LDP	-0.569	0.602	0.566	0.067	4.810	0.792	0.382	2.207	0.374	13.026
LPD	0.890	0.398	2.436	0.308	19.234	0.973	0.311	2.647	0.403	17.366
DP	-0.785	0.645	0.456	0.016	12.858	2.238	**0.043**	9.375	1.076	81.722
Anxiety	0.088	0.221	1.092	0.948	1.259	0.015	0.818	1.015	0.891	1.157
Depression	0.130	**0.007**	1.139	1.037	1.252	0.120	**0.005**	1.127	1.037	1.226
Self-efficacy	0.045	0.646	1.046	0.864	1.265	-0.080	0.336	0.924	0.785	1.086
PSQI	0.566	0.008	1.762	1.161	2.674	0.429	0.025	1.535	1.055	2.235

Multiple categorical logistic regression was used, with trajectory grouping as the dependent variable, and comparisons were made with the low QOL group and the medium QOL group to the high QOL group (*n* = 24), respectively including baseline demographic characteristics, surgery-related information, and scores on each scale in the first year postoperative. β, regression coefficient; OR: odds ratio; CI: confidence intervals.

## Discussion

This study examined the trajectories of QOL changes among pancreatic cancer survivors one year after surgery, categorizing them into three distinct trajectory groups. It also explored the factors influencing assignment to different trajectory groups. In this study, over 70% of participants were classified into the group with consistently moderate or high quality of life, while approximately 30% were classified into the group with low quality of life. This finding was consistent with a previous study on quality of life among breast cancer survivors (*N* = 124) following completion of primary treatment, in which approximately 80% of participants were classified into the moderate or higher quality of life group^33^. Furthermore, the results of this study indicated that the trend in functional domain scores across all three groups was consistently upward, with a slight decline at 12 months, conversely, the trend in symptom domain scores for the corresponding groups was consistently downward, with a slight increase at 12 months. Thus, the trend in quality of life for all pancreatic cancer patients was one of continuous improvement, with a slight decline after 12 months, which was largely consistent with that observed in other studies^34,35^. However, this study indicated that for all pancreatic cancer patients, quality of life did not improve even one year after surgery. Furthermore, a similar dynamic pattern was observed across the group, continuous improvement from the early to mid-postoperative period (0–6 months), followed by a slight decline in all functional domains and a corresponding rebound in symptom domains at 12 months post-surgery. This phenomenon was largely consistent with findings from previous qualitative studies on the quality of life of pancreatic cancer survivors^36^, which was associated with common challenges faced by patients as they transition from the role of a patient under intensive medical care to a survivor bearing long-term self-management responsibilities, as well as with factors such as anxiety about recurrence arising from the follow-up gap following the conclusion of adjuvant therapy and the psychosocial stress involved in reintegrating into societal roles^37^. At the same time, this was also related to factors such as the high malignancy of pancreatic cancer itself, high postoperative recurrence rates, and low 5-year survival rates^38^.

Besides, this study found significant heterogeneity in the scores of postoperative pancreatic cancer patients across functional and symptom domains, revealing that the patient population was not homogeneous during the postoperative recovery process but exhibited substantial individual differences. This was consistent with the findings of a longitudinal study involving a one-year follow-up of patients with neuroendocrine tumors (*n* = 39), which concluded that disease severity and clinical heterogeneity influence changes in quality of life over time^39^. Furthermore, this heterogeneity stemmed from multiple factors, at the treatment level, tolerance to adjuvant chemotherapy, as well as age, preoperative nutritional status and functional status collectively modulated an individual’s ability to cope with the disease and recover^40,41^. Therefore, the findings of this study provided guidance for clinical practice. Firstly, there should be a shift from a homogeneous management model to individualized supportive care pathways based on risk assessment and precise stratification. It recommended that standardized scales (e.g. EORTC QLQ-C30) should be used during routine follow-up to systematically screen and stratify patients’ functional status and symptoms^42^. High-risk patients should be identified and provided with proactive support and resource referrals early in the post-discharge period. For patients with delayed functional recovery, intensive, individualized rehabilitation training and physical therapy should be initiated. For patients with a high symptom burden, referral to a palliative care team or symptom management clinic for multidisciplinary intervention was recommended^43,44^. Second, future research was recommended to designate the 9–12-month postoperative period as a critical clinical intervention window. During this phase, patients’ psychological and symptom burdens should be proactively assessed, and transitional guidance provided to facilitate smoother and more sustainable long-term recovery. And utilize advanced statistical methods to identify distinct functional-symptom phenotypes and explore the multidimensional determinants underlying them, thereby providing an evidence base for precision medicine in further study^45^.

Furthermore, among the factors affecting QOL trajectory patterns, depressive symptoms and sleep quality were identified as independent predictors of different QOL trajectories. First, depression scores was a key factor in distinguishing all subgroups. Compared with the high QOL group, for each 1-point increase in depression score, the risk of patients falling into the low or moderate QOL group increased by approximately 13%. This suggested that depression was not merely a concomitant symptom of declining quality of life, but a core driving factor, this hypothesis had been validated by a longitudinal study on pancreatic ductal adenocarcinoma (*n* = 114)^46^. Depression systematically steered patients’ recovery processes toward a more unfavorable trajectory by undermining their motivation to recover, hindering healthy behaviors, and exacerbating the perception of physical symptoms^47^.

Second, sleep quality was also a strong independent predictor, with an impact even more pronounced than that of depression. For every 1-point increase in the PSQI score, the risk of patients falling into the low QOL group and the moderate QOL group increased significantly by 76.2% and 53.5% respectively. This confirms the fundamental role of sleep disturbances in the recovery of pancreatic cancer patients^48,49^. Sleep disorders can directly lead to daytime fatigue, cognitive decline, and impaired emotional regulation, creating a vicious cycle that hinders physical recovery and exacerbates symptom distress, ultimately was correlated with a continuous deterioration of patients’ quality of life^50^. In summary, these findings indicated that the incorporation of routine depression screening and sleep assessment into postoperative follow-up protocols. For patients with relevant issues, timely referral for psychological intervention and cognitive behavioral therapy targeting sleep disorders is of great importance for improving overall recovery outcomes^51^.The risk factors identified in our cohort may help inform supportive care strategies, but given the single-center design, these findings should be validated in multi-center studies before broader application.

Finally, in this study, the effects of factors such as anxiety, self-efficacy, BMI, and occupation did not reach statistical significance. However, when a broader range of variables was included in sensitivity analyses, anxiety emerged as a major predictor. This showed that anxiety, depression, and sleep disorders co-occur at a high rate in this patient population^52^, and their complex interactions warrant further exploration in future. Regarding the data outlier observed for occupational factors that no self-employed individuals were classified in the low QOL group, while this resulted in an anomalous statistical estimate for the variable, it revealed that certain occupational characteristics, such as work autonomy and time flexibility may serve as powerful protective resources, sufficient to prevent this group from falling into the worst quality of life trajectory. Given the limited sample sizes within subgroups, particularly for occupational stratification, these subgroup findings should be considered hypothesis-generating and exploratory rather than definitive or confirmatory.

However, patients who died or were lost to follow-up were excluded from the final analysis, which may have affected our trajectory estimations. It was a limitation of our study, which may introduce substantial survivorship bias. Excluding patients who died or were lost to follow-up introduces survivorship bias, meaning our trajectory analyses only represent patients who survived long enough to complete the required assessments. Given that pancreatic cancer is associated with early postoperative mortality, our estimates likely reflect a healthier subset of the original cohort. Consequently, our reported QOL trajectories are probably more favorable than the true trajectories of the entire patient population, including those who died early or became too ill to participate. We emphasized that our findings apply primarily to patients who survive the early postoperative period and are well enough to provide longitudinal PRO data. And suggested that future studies consider joint modeling of longitudinal QOL and time-to-event outcomes to reduce this bias, or employ inverse probability weighting to account for differential dropout due to death.

A strength of this study was its use of a flexible, person-centered statistical approach, which can adaptively capture individual differences within the pancreatic cancer patients who underwent surgery that exhibit similar response patterns over time. Besides, by identifying factors influencing QOL trajectories and focusing on variables measured after surgical treatment for pancreatic cancer patients, the study identified high-risk groups likely to experience a decline in quality of life. Though longitudinal quality of life (QOL) assessments have been conducted in pancreatic cancer, and trajectory-based analyses are well established in breast and other cancers, their application in pancreatic cancer—particularly using patient-reported outcome (PRO)-based longitudinal data with repeated measurements across the disease continuum remains relatively limited. Furthermore, we emphasize that our study is among the first to identify distinct QOL trajectory patterns specifically in a pancreatic cancer cohort undergoing modern treatment paradigms, and to examine clinical and demographic predictors associated with these trajectories.However, given the following limitations, the results should be interpreted with caution. First, this study included only pancreatic cancer surgery patients selected via convenience sampling from a single medical center in China, which limited the generalizability of our findings and any derived clinical implications. Practice patterns, patient demographics, healthcare access, follow-up intensity, and supportive care resources may differ substantially across institutions and countries. Therefore, the clinical implications discussed herein should be considered specific to our setting and should not be broadly generalized without external validation. Second, the study ultimately included only 104 participants for trajectory analysis, the sample sizes of the low QOL group and high QOL groups accounted for less than 30% of the total sample, and there were insufficient measurement time points within one year after surgical treatment, which may have affected the shape of the trajectories^33^. Third, without adjusting for response bias, significant changes in health-related quality of life (HRQOL) may be overestimated or underestimated. Therefore, future study can focus on whether response bias represents a potential source of undesirable bias. Fourth, the study used SAS and SDS scales for assessing anxiety and depression which focus on patient-reported outcomes (PRO) in the context of QOL. The scales have been extensively used in clinical research in China, providing a rich body of normative and disease-specific reference data. However, this may affect the adequate reflection of anxiety and depression symptoms in pancreatic cancer survivors when instead of the internationally recognized Hamilton Scale. Finally, patients were recommended to follow a standard postoperative surveillance schedule. However, as is common in real-world clinical settings, not all patients adhered strictly to this schedule or completed all planned evaluations at our center. Additionally, due to individual differences among pancreatic cancer patients, variations in chemotherapy tolerance, and drug sensitivity, the chemotherapy regimens are not standardized, making it difficult to monitor the follow-up completion rates and disease-free survival. The results of this study indicated that over 70% of patients remained recurrence-free during the follow-up period. Univariate analysis showed that recurrence status was not a factor influencing quality of life. Therefore, the reasons for this phenomenon cannot yet be fully explained. Consequently, future multi-center cohorts or randomized controlled trials or studies employing causal inference methods are needed to establish causal relationships between the examined factors and QOL trajectories.

## Conclusions

This longitudinal study revealed heterogeneous recovery trajectories in quality of life of pancreatic cancer patients during the first year after surgery, which can be systematically categorized into high, moderate, and low groups. A common pattern emerged, all patients experienced a slight decline in quality of life at 12 month after surgery, highlighting the particular challenges of transitioning from patient to survivor roles. Depressive symptoms and sleep quality are associated with patients’ quality-of-life trajectories. These findings advocated shifting clinical practice from homogenized management towards risk-stratified precision support models, incorporating routine psychological and sleep screening and interventions into standard care. Proactive transitional guidance should be provided during the critical 9–12 month after surgery window. This study provided evidence for implementing the biopsychosocial model in pancreatic cancer care. Future further studies and interventional trials were required to validate these findings and translate them into clinical practice, thereby comprehensively enhancing patients’ long-term quality of life.

## Supplementary Information

## Data Availability

All data are available upon request to the corresponding author.
