# Supplementary material for: Trajectories of quality of life in pancreatic cancer survivors during the first year after surgery: a longitudinal study
Source: Sci Rep. 2026 Jun 16;16:27591. doi: 10.1038/s41598-026-58311-6 (PMC13538500; doi:10.1038/s41598-026-58311-6)
Supplement: Supplementary file 1 [file 41598_2026_58311_MOESM1_ESM.docx]

**1.Supplementary Analysis**

**1.1 Supplementary Trajectory Plots and Captions for the Three Trajectory Models**

**Figure 2 Trends in function, symptom scores (standardized) for the three different trajectories(n=104)**

Group 1: High quality of life group; Group 2: Moderate quality of life group; Group 3: Low quality of life group; The shaded area represents the 95% confidence interval for each trajectory group score;The scores of functional domain and symptom domain were standardized by Z-scores. Higher standardized scores in the functional domain indicate better quality of life, while lower standardized scores in the symptom domain indicate better quality of life.

**1.2 Supplementary Evaluation Metrics for the Classification Capabilities of Two Trajectory Models**

The classification quality of the Class 2 and Class 3 nonlinear trajectory models (ng2_d2, ng3_d2) was evaluated. The results show that the mean maximum a posteriori probabilities for the three trajectory groups in the ng3_d2 model were 0.918 ± 0.125, 0.951 ± 0.062, and 0.920 ± 0.148, respectively, with medians of 0.997 (0.837, 1.000), 0.975 (0.943, 0.989), and 0.998 (0.958, 1.000); In the ng2_d2 model, the mean maximum a posteriori probabilities for the two trajectory groups were 0.956 ± 0.083 and 0.926 ± 0.138, respectively, with medians of 0.989 (0.962, 0.999) and 0.999 (0.948, 1.000). The proportion of patients with a maximum a posteriori probability below 80% was low in all groups, suggesting that both models demonstrated good accuracy in trajectory grouping.

**Table3 Classification quality of trajectory models**

| **Model** | **n** | $\boldsymbol{P}_{\boldsymbol{max}}$**<80%** | ${\bar{\boldsymbol{P}}}_{\boldsymbol{max}}$**±SD** | **Median(Q1, Q3)** |
| --- | --- | --- | --- | --- |
| **ng3_d2** |  |  |  |  |
| high QOL group | 24 | 0.17 | 0.918±0.125 | 0.997(0.837, 1.000) |
| moderate QOL group | 51 | 0.04 | 0.951±0.062 | 0.975(0.943,0.989) |
| low QOL group | 29 | 0.17 | 0.920±0.148 | 0.998(0.958, 1.000) |
| **ng2_d2** |  |  |  |  |
| high QOL group | 79 | 0.06 | 0.956±0.083 | 0.989(0.962, 0.999) |
| low QOL group | 25 | 0.16 | 0.926±0.138 | 0.999(0.948, 1.000) |

1. ${\bar{\boldsymbol{P}}}_{\boldsymbol{max}}$ mean maximum posterior probability; SD, standard deviation; Q1, 25th percentile; Q3, 75th percentile.

2. Patients with Pmax < 0.8 refers to the proportion of patients whose maximum posterior probability of belonging to the assigned trajectory class is less than 0.8.

3. All mean values and median values are calculated based on individual-level posterior probabilities of assignment.

4. ng3_d2 and ng2_d2 refer to the 3-class and 2-class nonlinear trajectory models, respectively.

**1.3 Comparison of Trajectory Models Using the LMR-LRT**

Table 4 presents pairwise comparisons of models across different trajectory groups under linear and nonlinear assumptions, based on the main analysis and supplemented with the LMR-LRT. The results of the LMR-LRT test indicate that, in both linear and nonlinear trajectory models, the k+1-group model is significantly superior to the k-group model (P < 0.05), further supporting the three-category trajectory grouping.

**Table4 Effectiveness of multivariate trajectory model fitting for standardized function, symptom scores**

| **ng** | **AIC** | **BIC** | **Ek** | **LMR-LRT**  **test *P*** | **Avepp** | | | | | **Class Proportion(%)** | | | | |
| --- | --- | --- | --- | --- | --- | --- | --- | --- | --- | --- | --- | --- | --- | --- |
|  |  |  |  |  | **1** | **2** | **3** | **4** | **5** | **1** | **2** | **3** | **4** | **5** |
| Linear | | | | | | | | | | | | | | |
| 2 | 1629.16 | 1689.62 | 0.82 | **<0.001** | 0.96 | 0.92 |  |  |  | 75.96 | 24.04 |  |  |  |
| 3 | 1565.36 | 1658.07 | 0.81 | **<0.001** | 0.91 | 0.93 | 0.94 |  |  | 49.04 | 32.69 | 18.27 |  |  |
| 4 | 1535.62 | 1656.54 | 0.78 | **<0.001** | 0.94 | 0.93 | 0.88 | 0.93 |  | 59.62 | 19.23 | 12.5 | 8.65 |  |
| 5 | 1533.6 | 1686.76 | 0.82 | **<0.001** | 0.94 | 0.91 | 0.9 | 0.94 | 0.99 | 56.73 | 20.19 | 12.5 | 4.81 | 5.77 |
| Quadretic | | | | | | | | | | | | | | |
| 2 | 1597.86 | 1674.44 | 0.84 | **<0.001** | 0.96 | 0.93 |  |  |  | 75.96 | 24.04 |  |  |  |
| 3 | 1496.8 | 1613.69 | 0.81 | **<0.001** | 0.92 | 0.95 | 0.92 |  |  | 23.08 | 49.04 | 27.88 |  |  |
| 4 | 1483.85 | 1632.98 | 0.77 | **<0.001** | 0.96 | 0.9 | 0.92 | 0.89 |  | 54.81 | 21.15 | 14.42 | 9.62 |  |
| 5 | 1484.8 | 1678.27 | 0.86 | **<0.001** | 0.94 | 0.91 | 0.94 | 0.97 | 1 | 55.77 | 20.19 | 14.42 | 5.77 | 3.85 |

ng: number of trajectory group sets; d:1 means linear function, 2 means nonlinear function with square term; AIC, BIC: Akaike Information Criterion, Bayesian Information Criterion, the smaller means the better the model fitting; Ek: entropy value, the larger means the better the quality of trajectory classification; Class Proportion. LMR-LRT test: Lo-Mendell-Rubin Likelihood Ratio Test, used to compare the goodness of fit between k-group and (k+1)-group models; a *P*-value<0.05 indicates that the (k+1)-group model fits significantly better than the k-group model(k:2~5).

**1.4 Sensitivity Analysis Using Two Trajectory Groups**

To verify the robustness of the main analysis results, this study further conducted a sensitivity analysis using two nonlinear bivariate trajectory models (ng2_d2) that include quadratic terms. We re-examined the characteristics of quality-of-life trajectories, differences between groups, and influencing factors to confirm the reliability of the main study’s conclusions.

**1.4.1 Trajectory Plots**

**Figure 1 Trends in function, symptom scores (standardized) for the two different trajectories(n=104)**

Group 1: High quality of life group; Group 2: Low quality of life group; The shaded area represents the 95% confidence interval for each trajectory group score. The scores of functional domain and symptom domain were standardized by Z-scores.Higher standardized scores in the functional domain indicate better quality of life, while lower standardized scores in the symptom domain indicate better quality of life.

**1.4.2 Analysis of Differences in Characteristics Among Different Trajectory Groups**

The results of the between-group analysis for the two trajectory groups showed that there were statistically significant differences (P < 0.05) in the mean scores between the high-quality-of-life group and the low-quality-of-life group for the Self-Rating Anxiety Scale, the Self-Rating Depression Scale, the Self-Efficacy Scale, and the PSQI scores, consistent with the findings of the main analysis. Regarding BMI and occupation, the differences among the three trajectory groups were statistically significant, but the differences between the two trajectory groups were not significant.

**Table 1. Comparison of differences among different trajectory groups(n=104)**

| **Variable** | **Total** | **Traj Group** | | **Statistic** | ***P*** |
| --- | --- | --- | --- | --- | --- |
|  |  | **high QOL group (n=79)** | **low QOL group (n=25)** |  |  |
| Sex, n (%) |  |  |  | χ²=0.033 | 0.856 |
| Male | 64 (61.54%) | 49 (62.03%) | 15 (60.00%) |  |  |
| Female | 40 (38.46%) | 30 (37.97%) | 10 (40.00%) |  |  |
| Age Group,year, n (%) |  |  |  | χ²=2.914 | 0.233 |
| ~59 | 56 (53.85%) | 42 (53.16%) | 14 (56.00%) |  |  |
| 60~70 | 24 (23.08%) | 21 (26.58%) | 3 (12.00%) |  |  |
| 71~ | 24 (23.08%) | 16 (20.25%) | 8 (32.00%) |  |  |
| BMI, n (%) |  |  |  | - | 0.059 |
| Underweight | 67 (64.42%) | 54 (68.35%) | 13 (52.00%) |  |  |
| Normal weight | 27 (25.96%) | 16 (20.25%) | 11 (44.00%) |  |  |
| Overweight | 10 (9.62%) | 9 (11.39%) | 1 (4.00%) |  |  |
| Occupation, n (%) |  |  |  | - | 0.271 |
| Civil Servant | 5 (4.81%) | 3 (3.80%) | 2 (8.00%) |  |  |
| Retired | 66 (63.46%) | 49 (62.03%) | 17 (68.00%) |  |  |
| Staff | 19 (18.27%) | 15 (18.99%) | 4 (16.00%) |  |  |
| Freelancer | 9 (8.65%) | 9 (11.39%) | 0 (0.00%) |  |  |
| Others | 5 (4.81%) | 3 (3.80%) | 2 (8.00%) |  |  |
| Surgical Method, n (%) |  |  |  | - | 0.239 |
| PD | 41 (39.42%) | 31 (39.24%) | 10 (40.00%) |  |  |
| LDP | 20 (19.23%) | 17 (21.52%) | 3 (12.00%) |  |  |
| LPD | 27 (25.96%) | 17 (21.52%) | 10 (40.00%) |  |  |
| DP | 16 (15.38%) | 14 (17.72%) | 2 (8.00%) |  |  |
| Educational Attainment, n (%) |  |  |  | - | 0.432 |
| Junior High School and Below | 44 (42.31%) | 34 (43.04%) | 10 (40.00%) |  |  |
| Senior High School | 30 (28.85%) | 25 (31.65%) | 5 (20.00%) |  |  |
| University | 13 (12.5%) | 8 (10.13%) | 5 (20.00%) |  |  |
| Postgraduate and Above | 17 (16.35%) | 12 (15.19%) | 5 (20.00%) |  |  |
| Type Of Medical Insurance, n (%) |  |  |  | χ²=0.000 | 0.996 |
| Employee Medical Insurance | 79 (75.96%) | 60 (75.95%) | 19 (76.00%) |  |  |
| Non-local Medical Insurance | 25 (24.04%) | 19 (24.05%) | 6 (24.00%) |  |  |
| Surgical Method2, n (%) |  |  |  | χ²=3.106 | 0.078 |
| PD&LPD | 68 (65.38%) | 48 (60.76%) | 20 (80.00%) |  |  |
| DP&LDP | 36 (34.62%) | 31 (39.24%) | 5 (20.00%) |  |  |
| Relapse status |  |  |  | χ²=2.402 | 0.121 |
| No | 75(72.12%) | 60(75.95%) | 15(60.00%) |  |  |
| Yes | 29(27.88%) | 19(24.05%) | 10(40.00%) |  |  |
| Anxiety, Mean ± SD | 44.10±8.37 | 42.06±7.26 | 50.55±8.49 | t=-4.892 | **<0.001** |
| Depression, Mean ± SD | 44.81±10.36 | 43.48±10.44 | 49.00±9.09 | t=-2.373 | **0.020** |
| Social support, Mean ± SD | 39.21±6.87 | 39.58±7.14 | 38.04±5.93 | t=0.978 | 0.331 |
| Self-efficacy, Mean ± SD | 23.27±5.50 | 24.14±5.71 | 20.52±3.69 | t=3.701 | **<.001** |
| PSQI, Mean ± SD | 12.06±2.60 | 11.56±2.47 | 13.62±2.40 | t=-3.655 | **<.001** |

1. Mean±SD: Mean ± standard deviation (n=sample size); Median: median; (Q1, Q3): interquartile range; Z: Wilcoxon rank-sum test; χ²: Chi-square test; -: Fisher's exact test. t: t test.

2. Categorical variables were presented as number (percentage). For continuous variables, the mean and standard deviation were rounded to two decimal places, and the *P* value was rounded to three decimal places. Continuous variables with normal distribution and homogeneous variance were expressed as Mean±SD, while those without were expressed as Median (Q1, Q3)

**1.4.3 Multivariate Logistic Regression Analysis**

Variables with P < 0.1 in the univariate analysis were included in the multivariate analysis. The results showed that anxiety was an independent predictor of quality of life in pancreatic cancer patients (P < 0.05), whereas in the primary analysis, depression and PSQI scores were the independent predictors of quality of life in pancreatic cancer patients.

**Table2 Determinants of different quality-of-life trajectory patterns(n=104)**

| **Variable** | **β** | ***P*** | **OR** | **OR 95%CI** | |  |
| --- | --- | --- | --- | --- | --- | --- |
| BMI(Reference：Normal weight) |  |  |  |  |  |  |
| Underweight | 1.135 | 0.056 | 3.112 | 0.979 | 10.362 |  |
| Overweight | 0.165 | 0.891 | 1.179 | 0.055 | 9.624 |  |
| Surgical Method2(Reference：PD&LPD) |  |  |  |  |  |  |
| DP&LDP | -0.562 | 0.384 | 0.570 | 0.148 | 1.947 |  |
| Anxiety | 0.094 | 0.033 | 1.099 | 1.011 | 1.205 |  |
| Depression | 0.037 | 0.201 | 1.038 | 0.982 | 1.101 |  |
| Self-efficacy | -0.025 | 0.722 | 0.975 | 0.844 | 1.117 |  |
| PSQI | 0.200 | 0.093 | 1.222 | 0.971 | 1.558 |  |

1. β, regression coefficient; P, significance level; OR, odds ratio; 95%CI, 95% confidence interval.

2. Multivariable binary logistic regression was performed with trajectory group as the dependent variable and the low quality-of-life group as the reference.

3. Variables with *P*＜0.1 in univariate analysis were entered into the model. All assumptions of logistic regression were met.
